# A Pill in the Lifeworld of Women in Burkina Faso: Can Misoprostol Reframe the Meaning of Abortion

**DOI:** 10.3390/ijerph16224425

**Published:** 2019-11-12

**Authors:** Seydou Drabo

**Affiliations:** Institute of Health and Society, Faculty of Medicine, University of Oslo, 0315 Oslo, Norway; draboseyd@yahoo.fr

**Keywords:** misoprostol, women, abortion, ethnography, Burkina Faso

## Abstract

In Burkina Faso, induced abortion is socially stigmatized, condemned, disapproved and legally restricted to cases of rape, incest, fetal malformation or endangerment to the life of the mother. Many women often resort to unsafe procedures to induce abortion, which puts their health at great risk. Misoprostol, which is officially restricted to the treatment of postpartum hemorrhage or post-abortion care, is also used illegally by women to terminate their pregnancies. Misoprostol represents an addition to the existing abortion methods, such as vacuum aspiration, which health workers have often used to induce abortion clandestinely. Many women also use misoprostol to self-induce abortions, replacing abortifacients such as herbal teas, potions, high doses of antimalarial drugs, or bleach. Despite the changes that occur in abortion access due to the use of misoprostol, little is known about what the drug means to its users and how this meaning can in turn influence the meaning of abortion. The aim of this paper is to describe how the use of misoprostol to terminate pregnancy contributes to changing women’s perception of the meaning of abortion. This paper is based on ethnographic fieldwork conducted between March 2016 and February 2017 in the city of Ouagadougou, Burkina Faso. By examining the relation between the use of misoprostol and the meaning that women give to abortion, this study found that women experience abortion either spontaneously or using emergency contraception with misoprostol. Through the experience of women, this paper claims that the meaning of abortion should be seen as a social construct and fundamentally rooted in individual practices and experiences rather than being subject to dichotomist global discourse.

## 1. Introduction 

Abortion is a complex subject that has given rise to intense debate over its “definition” and its social, political, and legal status. In general, the needs of women are not always taken into account during these debates [1]. Hence, the relevance of looking at the meaning that they give to an “interruption” of pregnancy, because their attitude towards abortion could depend on the meaning given to that interruption. The aim of this paper is to describe how the use of misoprostol to terminate pregnancy contributes to changing women’s perception of the meaning of abortion and it experience. 

Misoprostol is a drug that entered in the global market in the late 1980s, and it was originally produced for the prevention of gastrointestinal ulcers [2]. Misoprostol is also used in obstetrics and gynecology to induce labor, to prevent and treat postpartum hemorrhage, and to manage spontaneous abortion [3]. It is also gaining dominance worldwide as a drug that can be effective and safe to end a pregnancy when used at the right time and with the right dosage under the supervision of trained health professionals [4]. Given that sub-Saharan African countries have the highest rates of maternal mortality worldwide due to the consequences of abortion and hemorrhage [5], public health actors have advocated for increasing the availability and accessibility of misoprostol in order to reduce maternal deaths [6]. Abortion in the second trimester of a pregnancy can be performed by the administration of misoprostol using a variety of dosages and routes of supply [7,8]. This includes diagnosing and dating the pregnancy, administering the drug following instructionson appropriate use [9]. For example, although misoprostol is described as a safe method to terminating a pregnancy, it uses requires an appropriately trained provider in order to meet safety [10]. Therefore, accessing misoprostol does not necessarily guarantee safe abortion if the modalities of it uses are not respected. Improper use of misoprostol can lead to life-threatening complications such as collapse, bleeding and fever [11]. Moreover, a recent report on the issue of abortion worldwide suggests that clandestine abortion in legally restrictive settings is becoming relatively safer as misoprostol is replacing harmful methods [12]. Since 2005, the World Health Organization [8] has recognized misoprostol as a life-saving drug and recommended it on the list of essential medicines, although only “where permitted under national law and where culturally acceptable” in recognition of the controversial nature of misoprostol for use in induced abortion [8]. This stipulates that misoprostol be subject to specific use according to the social and political contexts of countries. 

In Burkina Faso, induced abortion is socially stigmatized, condemned, disapproved [13] and legally restricted to cases of rape, incest, fetal malformation or endangerment to the life of the mother. Misoprostol is officially restricted to the treatment of postpartum hemorrhage and post-abortion care. Misoprostol also circulates informally in cities such as Ouagadougou and is often referred to locally as the ‘abortion drug’. Misoprostol represents an addition to the existing abortion methods, such as vacuum aspiration, which health workers have often used to induce abortions clandestinely. Many women also use misoprostol to self-induce abortions [13,14,15,16], replacing abortifacients such as herbal tea [17], potions, high doses of anti-malarial drugs, or bleach [4]. Misoprostol does indeed diversify abortion access sources, because its marketing in pharmacies and drug stores makes it possible to access the drug in secrecy [18]. Despite the changes that occur in abortion access due to the use of misoprostol, little is known about what the drug means to it users and how this meaning can in turn influence the meaning of abortion. 

This study fits into the perspective of anthropological studies on the use of misoprostol to induce abortion by looking at how the drug can reframe the meaning of abortion for some of its users [18,19,20]. For example, De Zordo [19] shows how, in Brazil, induced abortion was tolerated morally among women who use misoprostol to induce abortion. Hardon and colleagues [20] discussed how girls use misoprostol in the Philippines for menstrual regulation (a euphemism for early abortion). Drawing on insights from this body of literature, this paper will discuss how using a pill rather than an invasive procedure could change the meaning of induced abortion from the perspective of women. 

## 2. Materials and Methods 

This paper is based on ethnographic fieldwork conducted between March 2016 and February 2017 in Ouagadougou (Burkina Faso). I focused on women’s perspectives of and experiences in seeking out abortion, including medical abortion using misoprostol. I conducted participant observation in streets and their marketplace, interacting regularly with the various women and drug vendors. I observed pubs and the streets of Kwame Nkrumah, a main thoroughfare with many hotels, restaurants, pubs, frequented by men and women who work as waitresses or sex workers, often referred to collectively as “*les filles de nuits*” (girls of the night). This gave me opportunity to start conversation around the issue of abortion and to negotiate in-depth interviews with some of the women, who, because of their work, are at risk of unintended pregnancy, abortion and their consequences [21].

In order to understand the context of drug sales and the interactions between drug sellers and consumers, I conducted participant observations and informal discussions with street drug vendors by spending time with them in their marketplace. During events such as The Panafrican Film and Television Festival of Ouagadougou, the public authorities officially establish a merchant street where traders come to expose their products. Drug vendors participate in these activities by exposing and selling their product for one week. I stayed in the store of one of the drug vendors who was selling non-Western contraceptive methods and other drugs purchased by women. The long hours spent waiting there (3 to 6 h) gave me both an opportunity to talk with both the drug vendor and some of his clients. 

In addition to participant observation, I conducted in-depth interviews with 46 women (in the perspective of the broad PhD research) about their reproductive trajectories, their perceptions and practices of contraceptive methods and abortion drugs and the decision-making processes and networks involved in the procurement of misoprostol. Health care workers assisted me in identifying women who had sought family planning and post-abortion care and who were willing to participate. I used my social network to identify other participants from the general population. This consisted of asking women who were more or less close to me (neighborhood, former classmates, friends, etc.) and who agreed to participate in this study. Subsequently, I relied on these women to access other women (friends of my friends, etc.) who were also willing to participate in this study. Of the 46 women I interviewed, 16 reported that they had undergone induced abortions in the past. These 16 interviews form the basis of the analysis in this paper. Apart from two women, I met in post-abortion care services, the rest of the women (14) I met outside of a health center. Among this group, nine were single women, four were co-habiting with a man, two were married and one was widowed. Seven of these women were working as servers in a pub, three were students, three were petty traders, one was a maid, one a housewife and one a public servant. Five of the women in this group had had more than one abortion. Together, these 16 women had 23 abortions between 2010 and 2017. Ten of them reported using misoprostol to terminate one or more pregnancies (five women used it vaginally with the support of the abortion provider and six women used it orally themselves or after receiving instruction from abortion providers or friends), while other methods included manual vacuum aspiration potassium permanganate, Chinese pills and recipes made from plants. The ten women who reported using misoprostol for abortion did so on pregnancies ranging from 1½ to 4 months. Among these women, one used the drug without confirming the pregnancy while the other one did not know how far along she was in her pregnancy at the time of using misoprostol. Seven out of the 16 women’s interviews are used in this paper because their cases are more illustrative of women’s abortion experiences and related issues.

### 2.1. Data Analysis and Ethical Issues

I conducted interviews in French, Mooré or Dioula depending on participants’ preferences. I also fluently speak the three languages. I tape-recorded the interviews (except 3 of them who were more comfortable with note taking) and they were transcribed verbatim. Interviews lasted between 20 min and one hour and 30 min. A research assistant transcribed the interviews and those recorded in Mooré or Dioula were translated into French. I did the final editing, checking all the transcripts in order to ensure accurate transcription and translation. 

The data analysis process was guided by a thematic approach that was both deductive and inductive [22]. The data was reviewed with certain preconceived categories derived from previous studies and from my own research experience [1,13,23]. For examples, theme such as women’s reproductive experience, affective trajectories, relationships with contraception and abortion technologies and the decision-making processes in relation to these methods are illustrative of the deductive analysis. The inductive analysis concerns themes that emerge directly from the data using inductive coding. In this framework, the transcripts were carefully read in order to identify the emerging themes. Phrases and sentences related to abortion experience and the way they define or perceived it were coded in the margins of the transcript sheets.

The themes identified in the research drew out historical details of the women’s lives, while pointing out the facts, practices or events that dealt with their reproductive life. The events were related to each other by taking into account their chronology. The approach makes it possible to see, for example, when and how the prevention, the occurrence and the termination of a pregnancy occurred in the person’s life; the motivations and facts that contributed to the occurrence of these events, their perception and so on. This data summarized the stories of women. 

Cross checking research material (interviews, field notes, and observation) and the summary of women’s stories allowed me to write a “problematized” portrait, i.e., a portrait of a research participant around an issue they experienced and described based on a specific context [24].

I used some of the portraits and excerpts from interviews with women that have used misoprostol to terminate a pregnancy to illustrate my findings. The quotes that I have chosen to use to illustrate the findings have been translated from French to English. I obtained ethical approval for this study from the Ethical Committee of Burkina Faso and the Norwegian Centre for Research Data. I read out loudly informed consent forms to the research participants, who provided oral consent by their own wish. Oral consent was also the most suitable because of the sensitivity of the topic for both health care providers, drug vendors and women. Despite reading informed consent, I tried to remind my participants of my good intentions. For example, I made women understand “My goal is not to judge but to understand...”. This attitude allowed me to establish a climate of trust with participants so that they would feel comfortable talking about their reproductive life experiences. I told all participants they could withdraw from this study at any time or choose not to participate in this study. The names of participants cited in the quotes are pseudonyms.

### 2.2. Abortion Debate and the Role of Technology 

Abortion remains a moral issue that raises debate that is usually framed as a battle between the fetus’s right to life and the woman’s right to choose [25]. At this level, different conflicting points of view are clear: one position maintains that the fetus is a life and claims that abortion should be criminalized [26]. Another position counters this argument by asserting that the fetus is not a life and that policy must be directed toward protecting a woman’s ability to control her own body by letting her choose whether to have an abortion or to carry a pregnancy to term [26]. The players in this debate involve a list of non-exhaustive actors, including religious actors, less well-known opponents of abortion, feminist groups, medical circles, family planning agencies, etc. [25,27]. Beyond discussion about the status of the fetus and the rights of women, debate also concerns a struggle over what the goal for abortion policy should be [27]. At this level, the debate focuses on different issues ranging from the recognition of abortion as a public health problem to the recognition of abortion as a woman’s right [1]. 

The issue of abortion as a public health problem has been addressed at various conferences and has been focused on the health consequences of unsafe abortions considered as major public health problems. Unsafe abortion, described as a cause of maternal morbidity and mortality in countries where abortion is illegal [28], has introduced discussions on the decriminalization of abortions. However, opponents of the decriminalization of abortion hardly recognize the link between illegality and the associated risks. For the latter, decriminalization would increase the occurrence of unsafe abortions, despite recent scientific evidence that has shown that the prevalence is low and relatively stable in countries where abortion is legal [29] in contrast to countries where abortion is illegal [1]. Moreover, given the lack of global consensus, emphasis has been placed on the prevention of abortions through universal access to family planning services, post-abortion care and the need for governments to guarantee individuals the exercise of sexual and reproductive rights. 

Regarding abortion as women’s rights, none of the international conferences have admitted a right to abortion and referred the decision back to the national authorities of countries [30]. As a result, in many countries, the legal status of abortion is more about health concerns than rights, as claimed by feminist movements since the 1960s. 

Furthermore, technology has not remained on the sidelines of the abortion debate. Callahan [31] described in five points how scientific development could reframe abortion debate through several implications [31]. First, the scientific developments have legal implications, as they can be significant in undercutting important factual assumptions underlying earlier court decisions. Second, the developments may have psychological implications, because new evidence may motivate people to think in different ways about their beliefs. Third, the scientific developments can have social implications in placing abortion in a different social context. Fourth, the developments may have political implications by serving as effective political capital if cleverly deployed. Fifth, the developments can have moral implications by prodding people to examine their consciences or by provoking new moral arguments. 

In practical terms, technological changes, such as fetal photography, ultrasound, advances in care for preterm infants, and fetal surgery, have facilitated personification of the fetus and challenged previous constructions of boundaries between fetus and infant [31,32]. The antagonist groups have used these different technologies in order to strengthen their positions. For example, the actors who think that women have the basic human right to decide when and whether to have children have debated the relevance of appropriate gestational age limits [32], while actors advocating against abortion have helped to shape this debate by using fetal images and by interpreting them in ways that suggest abortion is equivalent to murder [33]. As we can see, technology is making the abortion debate more complex, because different groups of people exploit it to support their perspectives, which contributes to perpetuation of the antagonistic positions. However, the complexity of the abortion debate involves historical and cultural meanings specific to each country [27]. 

### 2.3. Understanding the Social Context and the Illegal Circulation of Misoprostol 

The Burkinabe context offers a complex landscape for studying the use of misoprostol to terminate pregnancy. Burkina Faso is a sub-Saharan African country located in West Africa. The average age at first marriage is 17.9 years old, with nearly one-third of girls married between 15 and 19 years old and two-thirds married by the time they turn 24 years old [34]. The fertility rate was estimated at 5.71 children per woman by the World Bank in 2017. In Burkina Faso, sexuality in marriage is expected as elsewhere. However, sex also occurs frequently outside of marriage [35]. This increases the risk of unintended pregnancies. According to Bankole and colleagues [4], one-third of all pregnancies each year in Burkina Faso are unintended, and one-third of unintended pregnancies are ended by abortion. In rural areas, the abortion rate is slightly lower (22 per 1000 women) as compared to that in urban areas (42 per 1000). 

The practice of abortions, like in many societies [36], is subject to social disapproval in Burkina Faso. Abortion experiences are intimate and confined to silence [35]. Many women often resort to unsafe procedures to induce abortion, which can create great risks to their health. In general, half of women who induce their own abortions are estimated to experience complications as compared with approximately two in 10 women who go to health care providers [4] offering illegal abortions. The government’s principal policy response to the issue of unsafe abortions has been the implementation of a post-abortion care policy to treat the complications of unsafe abortions [30,37] through manual vacuum aspiration and misoprostol. 

My fieldwork focused on Ouagadougou, a city of 2.7 million inhabitants [38], which is also Burkina Faso’s administrative and economic center. In Ouagadougou, there is no access to abortion care in the public sector, except in the circumstances stipulated by law. Comprehensive post-abortion care services are offered in secondary and tertiary health care facilities and in some primary health care facilities in the public sector as well as in certain private sector and Non-Governmental Organizations facilities [39]. Misoprostol can be purchased for reproductive health indications by prescription in hundreds of pharmacies in the city [40]. There are mechanisms in place in health facilities to prevent misoprostol from being used outside the framework of the management of incomplete abortions and in the treatment of postpartum hemorrhage (Ouattara et al. 2019 forthcoming). However, the permissiveness of the drug distribution system in Burkina Faso that allows individuals to access the drug without showing a prescription generates conditions that favor illegal abortion [16]. In this context, the circulation of misoprostol obeys the same logic of drug exchange in developing countries where Van der Geest and Whytes [41] have shown that it often circulates as a commodity that can be sold and purchased [41]. Drug vendors in pharmacies, health workers and sex workers are among the actors involved in the network that enables women to access misoprostol illegally (Drabo 2019 forthcoming). 

### 2.4. Misoprostol Is Changing Access to Abortion 

The abortion experiences of women show that misoprostol has changed access to induced abortions, because it allows them to have abortions discreetly and at a relatively cheap cost as compared to other abortion methods, such as manual vacuum aspiration. Women described how misoprostol could be used at home, in a pub, guesthouse or any place to induce abortion. Here, 23-year-old Awa (single) explains her experience when she resorted to misoprostol to terminate a three-month pregnancy: 

“My boyfriend contacted a doctor who gave us an appointment in front of a guest house. My boyfriend paid for the room and then waited outside. I went inside with the doctor who put a white pill inside me. After that, I did not see him again. When I arrived home, I started bleeding a bit and it came out.” 

These examples show how misoprostol enables clandestine abortion services to work discretely, by removing the procedure from the health care setting where providers may risk prosecution if complications occur. In addition to meeting the need for discretion, misoprostol also changes the cost of abortion services. 

Women report that abortion with misoprostol was relatively affordable, around 15 USD, as compared to illegally induced abortions using manual vacuum aspiration, which can cost 45 USD. However, the price of misoprostol varies depending on how the product is accessed. It is relatively cheap when women procure it to have a self-induced abortion as compared to when they use a health worker. However, some women I spoke with had an abortion without paying money, because their acquaintances gave them misoprostol free. For example, Diane a 35-year-old woman lived with her partner for 17 years with whom she had two children. Her partner did not want another child. After she announced her third pregnancy, he asked her to have an abortion and threatened to leave her if she kept the pregnancy. Diane decided to contact one of her friends, a medical doctor, to obtain misoprostol, and he gave it to her free of charge. As she explained: 

“When I had my problem, I got the product for free. A friend helped me to get it from another friend. He did not buy it either, because they are both health professionals and they mutually support each other. The other friend could not refuse, because he knows that one day he may also need help (not only abortion) from my friend.” 

Furthermore, some of the research participants described how they obtained misoprostol from female friends or relatives who had used it to self-induce abortion. These female friends or relatives gave the remaining pills from the packet they purchased as a gift. Although she has never had an abortion, one woman in my study confided that she has misoprostol because her cousin gave her some tablets in case she would need them one day. She interrupted the discussion to locate the tablets and showed me a blister pack with six tablets missing. This fact of giving misoprostol means that the abortion of one woman can allow the abortion of another woman. 

In sum, misoprostol through the changes that it brings in terms of access to abortion is a response to the demands or the expectations of women seeking to terminate a pregnancy within the context of the restrictive abortion law in Burkina Faso. 

### 2.5. When Misoprostol Turns Induced Abortion to “Spontaneous Abortion”

Misoprostol plays an important role in the abortion experiences of women given that it has diversified pregnancy termination methods in addition to existing methods, such as manual vacuum aspiration and curettage. By describing their use of misoprostol and the abortion process, some of the participants, such as Adjara, stated that the drug interrupts pregnancy like a spontaneous abortion. 

Adjara is a 37-year-old woman who started using contraceptives at the age of 18 with the intention of avoiding pregnancy before marriage. During my interviews with her, she reported that a pregnancy before marriage would be unwelcome in her family because of their traditions and religious affiliation. As she said, “I come from a Muslim family and besides we have our tradition when you take a pregnancy automatically it is outside.” Later on, for fear of illness and being overweight, she decided to stop taking contraception after 4 years of use. Immediately after this cessation, she experienced three pregnancies before her marriage and decided to get rid of them. The first pregnancy was interrupted by a curettage performed by a health worker. She terminated the last two pregnancies herself using misoprostol, which she discovered through one of her acquaintances. Adjara declared that she used misoprostol the first time on a one-and-a-half-month pregnancy and for the second time, she did not confirm the pregnancy before using the drug. Comparing the two methods of abortion (curettage and misoprostol), Adjara said: 

“Since I discovered misoprostol I did not do curettage again. The curettage is very painful, and you do not know who is fiddling you. I do not know how to express it, but it is very painfully. While with misoprostol, you swallow and the next day you have small stomachache; then, you go to the toilet, you feel it goes down slowly, and then you take antibiotics after. It is a bit like having a spontaneous abortion...”. 

The example of Adjara shows that past abortion experiences can play an important role in the way women build their perceptions about abortion, because the fact of having experienced several abortions makes it possible to make comparisons between different episodes and methods. We see how Adjara refers to her three abortion experiences to state her preference for misoprostol over curettage. She justified her preference for misoprostol based on its process, which is less painful and interrupts pregnancy like a spontaneous abortion. Furthermore, Adjara’s reference to spontaneous abortion, even though she has not reported having had one, can express her attempt to describe a pregnancy termination with a positive note given that spontaneous abortions in Burkina Faso are morally accepted as compared to induced abortions. 

### 2.6. Misoprostol Is Like Emergency Contraception Pill 

The use of emergency contraceptives is part of the reproductive experience of women. In general, women use emergency contraceptives when they are in a situation that they consider to be at risk for pregnancy. Through their discourse during interviews and informal discussions, the use of emergency contraception, which they commonly call “norlevo”, is a common practice to avoid pregnancy, as testified by the following example of Veronique. Veronique is a 35-year-old woman living in a union with a man, and she was a mother of two children at the time of the interviews. She identified herself as a religious person, meaning she is practicing a religion (Protestant). Veronique used a contraceptive method; the first time was in 2012 after her second delivery by resorting to pills and injections. Afterwards, she stopped taking them, because she was not living in the same city with her partner. She said, “To take contraceptive methods is to say that we are sexually active. I don’t live with my husband, and I am not sexually active. So, I decided not to take contraception anymore.” However, Veronique confided in me that she has resorted to emergency contraception in case of sexual intercourse during a period in which she is likely to become pregnant. She said, “For us who are religious, it is acceptable, because nothing proves that there was going to be a pregnancy.” 

Furthermore, although most women I interviewed admitted to have used the morning-after pill at least once in their life, some of them found it ineffective in avoiding a pregnancy as explained by Nina, a 39-year-old woman who ironically declared: “Yes, I know I’ve used this before, but it does not work all the time. If you want take a pill of few seconds, it does not work.” Through this quote, Nina stresses that if there was a pill of some second unlike that of 72 h (morning-after pill), it will not work for everyone. Due to the uncertainties associated with the efficacy of the morning-after pill, some of the women I interviewed prefer to resort to misoprostol once they have a delay in the onset of menstruation: “Yes, because as soon as you know you have a delay, you take it.” These words of Adjara were confirmed during informal discussions I had with women in a maquis in the city of Ouagadougou. In this environment, the use of misoprostol is often trivialized, because the girls refer to the drug to tease each other. As explained by Flora (33 years old, living with a man) during informal discussion: “When you see a friend who is sad or who is looking sad, you propose to her to slip two tablets of miso in her beer in case there is a delay in her period that is worrying her” (laugh). 

As can be seen, the experiences and discourse of women shows that they tend to substitute misoprostol for an emergency contraception pill, especially since some of them do not hesitate to describe misoprostol by equating its mode of action with that of “norlevo” (emergency contraception). As does Flora, a single woman who during the interview has declared to having already resorted to misoprostol to stop a pregnancy (the pregnancy was two months): “If you know the effect of norlevo then you know the effect of misoprostol (Cytotec). When you take it after your dangerous period, it is only blood that you will see coming out later. …” (Flora, 33 years old, living with a man). 

Comparing misoprostol with emergency contraception pills makes one think about the following syllogism: if using emergency contraception does not mean having an abortion and misoprostol acts like emergency contraception, then the use of misoprostol does not necessarily mean you are having an abortion. Furthermore, it should be noted that women who tend to have a positive discourse on the use of misoprostol as a method of terminating a pregnancy have used it either on a confirmed pregnancy at an early stage (1 to 2 months) or after a delay in their menstrual cycle (2 weeks to 1 month). 

Examining the relation between the use of misoprostol and the meaning that women give to abortion, we see that women view an abortion either as spontaneous or as emergency contraception. This reference to spontaneous abortion and emergency contraception appears to be a way for women to confess their abortion with understatement in order to present a picture of abortion that is socially and legally acceptable. Indeed, in Burkina Faso, spontaneous abortion is considered a misfortune that can generate compassion for the “victim,” while emergency contraception reminds one of contraception, meaning a prevention of pregnancy before conception, which is also an issue that the women accept compared to abortion (which is a termination of the pregnancy after conception). The fact that they answer the question as to whether they have already voluntarily terminated a pregnancy by naming their experience shows that women do not deny that what they performed was an abortion, but they simply decide to present it in other way thanks to misoprostol and its mode of action. 

### 2.7. Women’s Abortion Experiences with Misoprostol 

The use of misoprostol for abortion is not equally available to all women. Indeed, women’s stories show that access to abortion is not a foregone conclusion. A forthcoming paper on the domestication of misoprostol by women highlights that terminating a pregnancy with misoprostol outside legal frameworks does not always turn out as expected. In this regard, some women can obtain medical abortion with misoprostol by resorting to private clinics. Women who do not have money to buy misoprostol are more likely to be victims of sexual harassment or sexual abuse from men that offer abortion services. Some women will perform self-abortion by administering drugs, but in most instances, they resort to misoprostol in contexts characterized by uncertainties related to a lack of information about the pregnancy and the drug. As illustrated by the case of Charlotte:

Charlotte, 26, is a student and mother of a 7-month-old child. After the birth of her child, charlotte chose not to use any contraceptive method because she feared the side effects. Resuming sexual intercourse, Charlotte said she was afraid of becoming pregnant because her partner refused to use condoms. After a two-month delay in her menstrual cycle, Charlotte thought she was pregnant despite the negative results of a pregnancy test she did herself. To remove doubt, she decided to terminate the “suspected” pregnancy. Charlotte decided to go to a pharmacy in the city center of Ouagadougou to obtain an abortion pill, she explained: 

“Once in the pharmacy. I told him that I have a young child and I realized that I am pregnant. It will be difficult for me to keep this pregnancy and to manage the other child. After my explanations, he told me there is a product that can terminate the pregnancy but without a prescription, he cannot sell it. However, he later reassures me that he can help me if I have money to buy the drug. The drug cost 11,000 of the West African CFA franc (XOF) (1 US equals approximatively 590 XOF). I did not have enough money that day and I had to return to the pharmacy 3 days later after getting money from a friend. The drug vendor gave me a tablet of misoprostol and told me that I should swallow four pills three times every four hours. However, before I left the pharmacy, he told me he is not responsible for any problem that will happen…”.

Charlotte’s experience shows her willingness to use misoprostol without being sure of being pregnant. In addition, she has access to the drug from the seller, who shows her a regimen without knowing her gestational age and his disengagement from the rest of the abortion process. It is clear that in these circumstances, the risk of the improper use of the drug becomes clear. Later in the discussion, Charlotte confessed that she resigned herself to not using the drug until the pregnancy becomes evident. Another woman, Samira, a 21-year-old woman (single) working in a pub, obtained misoprostol from a friend with minimal knowledge on the misuse of the product. During the interviews conducted with her, she confided that she was three months pregnant and decided to terminate it. She obtained misoprostol from a friend who did not tell her how to use it. She took the entire tray of pills at the same time (14 tablets). A few minutes later, she was admitted to hospital due to dizziness and pelvic pain. 

For other women, accessing abortion means experiencing failed abortion as illustrated by the case of Anna, a 30-year-old who became pregnant with her foreign boyfriend. She was not sure about how far along in the pregnancy she was but thought that it had been more or less three months since the baby was conceived. She worked as a hairdresser and earned between 15000 XOF to 20000 XOF a week. She supplemented her meagre income through sex work. She decided to terminate the pregnancy since she was concerned that her boyfriend would take the child to his home country. As she said: “I am not going to struggle to give birth to a child and they will come and take it from me one day”. When Anna was one month pregnant, she asked a friend to escort her to a woman who she knew that conducted abortions in her home. According to Anna, this abortion provider was not a health care worker, but learned how to conduct abortions after working with a health care worker. 

Anna and her friend visited the woman several times over a period of eight days before she finally agreed to help them. She asked them to pay 20,000 XOF, before placing a white product (I assumed it could be misoprostol) in Anna’s vagina and explaining that once Anna reached home, the fetus would be expelled. Unfortunately, the pregnancy was still intact after a week. After this failed abortion attempt, Anna was afraid to go back to see the woman because their previous meetings were difficult. With the help of the same friend, she decided to go to a clinic known to practice clandestine abortions three weeks later after the first attempt. Once she reached the clinic, the man who owned the clinic requested that she pay 2000 XOF for the examination. After the examination, he fixed the price of Anna’s abortion at 50,000 XOF and gave her an appointment for the same afternoon. Anna did not have enough money but decided to go to the appointment anyways with the intention of negotiating a discount. Once in the clinic, a secretary in the clinic discreetly informed Anna that she knew a place where Anna could have an abortion for less. Anna accepted the offer and was directed to the home of another woman. Anna did not know anything about the professional background of the woman, beyond that she offered abortion services. The woman performed Anna’s abortion in her house (abortion provider) by aspiration at a cost of 30,000 XOF.

Anna’s experience illustrates that accessing misoprostol outside legal frameworks may not guarantee access nor a successful abortion. Given the illegality of the abortion, in the case of failure, women are not able to hold the abortion providers accountable. Rather, they go to official health facilities to receive support for treatment if complications occur, or resort to another abortion provider until they are “satisfied”, such as in Anna’s case. In such circumstances, women may face unplanned expenses or experience delays in receiving the right care after starting the abortion process. 

## 3. Discussion

The aim of this paper was to describe how the use of misoprostol to terminate pregnancy contributes to changing women’s perception of the meaning of abortion. Based on ethnographic research, I have highlighted how the use of misoprostol allows women to access abortion at a relatively cheap cost, which corroborates with findings from other studies [7,42].

For women who have access to misoprostol, its use to stop a pregnancy influences how they define abortion, especially in view of the fact that misoprostol, as a technical object, can be a meaning-making vehicle [43]. That is to say, that it can impose a certain frame of thought [44]. By examining the relation between the use of misoprostol and the meaning that women give to abortion, this study has shown that, with misoprostol, women experience abortion either as spontaneous or as using emergency contraception. Thus, with misoprostol, the experience of abortion becomes different due to some of the following reasons: the early abortions women have allows them to see blood instead of a “constituted” fetus after expulsion; the process of pregnancy interruption is less painful, leading to a post-abortion period with less trauma; in addition, the fact of anticipating the use of misoprostol without confirming a pregnancy when they have a delay in their menstrual period allows them to maintain doubts about abortion and to equate the use of the drug to a contraceptive method. Furthermore, this reference to spontaneous abortion and emergency contraception allows them to present a picture of abortion that is socially, legally and morally acceptable. These findings confirm other anthropological studies [18,19] that have highlighted how misoprostol changes the meaning of abortion by making it a morally acceptable practice. The attitude of women reminds us that the meaning of abortion depends on the beliefs, perceptions and experiences of individuals. Thus, women’s “encounter” with misoprostol and its mechanisms of action help to “shape” their perception of abortion.

Furthermore, one of the major changes that misoprostol has brought to the abortion experience of women is the relative autonomy it guarantees from physicians and their instruments [45]. As with other abortion drugs, such as RU-486 (mifeprostol), misoprostol is a technology that allows women to be actors during the abortion procedure, which is in contrast with surgical methods that reduces them to the status of patients and dependent on medical actors to terminate their pregnancy [46]. This situation reduces the social and medical constraints that limit access to abortions (ibid). However, the context in Burkina Faso allows for some nuances, because women do not have equal access to abortion drugs, such as misoprostol. For example, a recent study in Ouagadougou has shown that the use of misoprostol is predominantly among women who have secondary and post-secondary education and who have high socioeconomic status [14]. Indeed, the introduction and use of misoprostol in Burkina Faso in the context of the restrictive abortion law gives opportunities to individuals with strong social networks and negotiating power to access misoprostol relatively easily, while those less powerful still struggle (Drabo 2019 forthcoming). Access to misoprostol through friends and drug vendors in pharmacies for abortion does not necessarily equate with quality, because the experience of women in this study and other studies has shown that the information provided by individuals working in pharmacies and drug shops is often poor, with few advising an effective regimen or not giving information on potential complications if they occur [47].

For poor women, the purchase and the use of misoprostol outside legal frameworks means risking sexual violence, misusing the product due to a lack of proper information with the risk of health complications, and experiencing inefficiency of the product when purchased from illegal abortionists, and the additional costs that this entails in the abortion-seeking process. These issues are in line with the findings of Zordo [19], who has shown in her studies in Brazil that the lack of legal access to misoprostol and to information on its safe use makes its purchase sometimes difficult and its use dangerous for low-income women. Despite accessing misoprostol, these women’s abortion experiences fall into the category of “less safe” abortion because of the lack of adequate information and support from trained individuals [10]. In addition, when abortion providers use their power to exploit women’s vulnerability by abusing them sexually, it is clear that for these women, the route to accessing a safer abortion, medically speaking, can be unsafe (Drabo 2019 forthcoming). The legal context of accessing misoprostol contributes to perpetuating forms of violence that undermine women’s sexual and reproductive health rights.

Understanding abortion experience, the risks associated with the use of misoprostol and inequalities in drug access could elicit policy changes by raising awareness of constraints related to the informal access of misoprostol and its health consequences. That is what makes this ethnography valuable.

This study has some limitations. The findings presented are from interviews with a relatively small group of participants recruited in the capital city and, thus, the views are not necessarily generalizable to all women. In particular, the majority of the women interviewed for this paper were recruited outside of health care facilities with the exception of two. The sample therefore is unlikely to represent the full spectrum of women’s abortion experience, particularly those who are admitted to hospital for post-abortion complications. 

## 4. Conclusions

Through the experiences of women cited in this paper, the meaning of abortion should be seen as a social construct and fundamentally rooted in individual practices and experiences. Rather than being subject to dichotomist discourses within the arena of global health. Furthermore, changes brought by misoprostol in term of access to abortion and the relative cheapness of abortion that results from the use of the drug make it likely that women will continue to resort to it. Providing women with knowledge about proper usage of the drug will be a relevant public health intervention that could reduce the health risks associate with the improper use of misoprostol. The Uruguay model could inspire this intervention by involving pharmacists/drug vendors and health workers in the process of evidence-based information on the safe use of misoprostol [48].
